# Thoracentesis

**Published:** 1878-03-20

**Authors:** Franklin Staples

**Affiliations:** Winona, Minn.


### THORACENTESIS.

### The Aspirator, the Trocar and Canula, or the Free Incision ?

By Franklin Staples, M.D., of Winona, Minn.

The New York *Record* of January 5th contained an interesting and practical article on thoracentesis, by Chas. W. Packard, M.D., of New York.

Dr. Packard gives four cases: First, that of a woman, aged 48, who had suffered with pleuritis ten days, and was dying from the immediate effects of a large pleuritic effusion. The fluid, which was serous (not purulent), was removed by aspiration. Great and immediate relief followed, and we are led to infer that recovery took place. The second case was that of a young married woman of very stout habit, admitted to the hospital six weeks after being attacked with pleuritis. It is stated that, when first seen, the patient was in a "bad way;" was moderately cyanotic; was constantly expectorating pus in immense quantities. Sixty-four ounces of pus were drawn off by aspiration; air rushed in through the opening that had been formed between the pleural cavity and the bronchial tubes, and pneumothorax took the place of the previous pyothorax. Moreover, the pus could not all be pumped out, and, although some temporary relief had been gained, shortly the symptoms were aggravated, and septicæmia threatened. A free incision was now made, the pus drawn off, and the cavity washed out through a flexible rubber tube with a weak solution of carbolic acid. This treatment was continued for a short time, and recovery resulted. The third case was that of a child, 7 years old, from whose chest nine ounces of pus were aspirated, and cure afterwards resulted in the case by the spontaneous evacuation of the pus through the lung and bronchial tubes. In the fourth case, a young man had been ill for two months. Seventy-two ounces of laudable pus was removed from the pleural cavity by aspiration. The patient was relieved, but in three or four weeks the pus had reaccumulated, and a free incision two inches long was made between the eighth and ninth ribs, and 100 ounces of pus were removed. The carbolized solution was injected daily; treatment continued for several weeks, and recovery took place.

The brief comments here submitted upon the excellent article of Dr. Packard are for the purpose of getting light on the following practical questions: In the operation of thoracentesis, shall we use the aspirator, according to Bowditch, or the trocar of Trousseau, or make the free incision with the bistoury? or, if different cases and conditions require different operative treatment, then what, if any, general rule shall guide us?

Dr. Packard says: "If repeated operations are necessary, it becomes a question whether aspiration should not give place to the operation by incision."

"If the secretion of pus is itself decreasing, and the constitutional disturbance is moderate, and especially in a young subject, we have reason to hope for an ultimate cure by aspiration. If, on the other hand, the pain and constitutional irritation continue severe, and the pus rapidly reaccumulates, I think there is no question that the pleural cavity should be opened by an incision large enough to admit of a ready evacuation of its contents, and of the manipulations necessary in washing it out, if the pus should be of an irritating quality."

Now, in place of the foregoing, I would offer the following amendment, making the line between the different operations to be more arbitrary, and as follows: If the contents of the pleural cavity is serum, I would always aspirate; but if it is pus, I would always use as large a trocar and canula as possible, or make a free incision with the knife, and then use the antiseptic wash, as described in the cases given above. In most cases the aspirator may be first used to test the character of the effusion. With our present facilities for the antiseptic treatment of disease, and for preventing infection in surgical operations, we are able to more than compensate for any evil resulting from the admission of air into the suppurating pleural cavity. In fact, the operation for the relief of pyothorax should be performed as much for the purpose of disinfecting the diseased cavity as for merely evacuating its contents.

Again, on the question of the time for operative measures to be resorted to, Dr. Packard's article has the following: "The time for interference must differ with differing cases. A latent pleuritic effusion, the result of a low grade of inflammatory action, and containing very little fibrin, while all the more likely to degenerate into pus, will not make many or strong adhesions, and, in this respect, may safely be left for weeks." [Query—What is the advantage of leaving such a case for weeks? Surely the risks of thus leaving it are greater than any incident to the early use of the aspirator?] "But," says the article, "an acute pleurisy is a different thing, and I think three weeks is the outside of safety against the formation of permanent and disastrous adhesions." [Query—Is it not well to avoid the outside limits, and relieve the oppressed absorbents while their action may yet be restored, and while the effusion is yet only serous in character?] As for evidence of the correctness of the views here given, both concerning the kind of operation and the best time for operative interference, the reader is referred to the history of every case given in Dr. Packard's article. I will add the brief notes of two cases, the history and results of which show the advantage of the use of the open canula and the antiseptic washings in advanced pyothorax.

I. A young man, 16 years old, the son of a farmer, presented himself for treatment in June, 1874, having ten weeks before sufferd from a severe attack of pleuro-pneumonia, while at work in the woods away from home. There was emaciation, with great exhaustion, dyspnœa, a rapid and feeble pulse, septicæmia, swelling of the feet, and the physical signs of an immense accumulation of fluid in the right pleural cavity. Paracentesis was performed by means of an aspirator, and five pints of feted pus drawn off, no air being allowed to enter the cavity. The operation was repeated four times in four weeks, and an average of four pints drawn each time. At the second and fourth operation blood was mingled with the pus in the receiver of the aspirator. The blood was fresh and readily coagulated. Any attempt to exhaust the gas from the cavity, by continuing to work the aspirator after the pus had ceased to flow, caused distress to the patient, and a renewal of the hemorrhage. Each operation in a measure relieved the more urgent symptoms of dyspnœa and septicæmia, but at the end of the month the patient was unable to move in bed unaided, suffered from hectic, was extremely emaciated, and was evidently failing. I now adopted the plan of drawing the pus through a large canula without the aspirator, and of performing the operation as often as every second or third day, and, after a short time, every day, allowing each time the free ingress of air. A very weak solution of carbolic acid in warm water was used as an injection at each operation, a small quantity of the wash being thrown in, and this drawn off, and then another injection made until the fluid drawn was quite clean. The patient was kept up on wine, iron, and quinine, and upon animal food, consisting largely of wild game. From the time of the change of treatment from the use of the aspirator to that of the simple canula, and of the more frequent performance of the operation, with the use of the antiseptic wash, there was steady improvement in the condition of the patient, but recovery was slow, and the adhesions were such as never to allow the lung to expand to any considerable extent.

At the end of eight months the thorax was an inch less in circumference on this side at the level of the nipple than on the other side, and a line through the chest, from the nipple backwards to the lower angle of the scapula, is at least an inch shorter than the same on the other side.

My notes at this time, eight months after the first aspiration, state that the canula is at present introduced morning and night, and about three or four ounces of purulent serum drawn at each time. A careful record of the amount of pus obtained, and which has varied in quantity at different times, has been kept, and the aggregate is upwards of 100 pints. Carbolic acid, chlorate of potassa, port wine, and the permanganate of potassa have successively been used in the injections. Cod-liver oil, with the phosphate of lime, has been given largely, together with quinine and the mineral acids. Three years and eight months have now passed, and there is yet a fistulous opening communicating with a small cavity. Of late a silver tube has been worn, allowing the slight discharge to take place constantly, and there is evidence of a more rapid diminution in the size of the cavity since the maintenance of this constant drainage. It is probable that an excision of a section of the rib at the place of the puncture, thereby allowing a more free and constant evacuation of the pus, would have hastened the process of cure.

II. Dr. M. Hagan, of St. Paul, Minn., reports a case in which he performed thoracentesis in a man who had been attacked with pleuro-pneumonia one month before. Two quarts of pus were drawn at the first operation with the trocar. A piece of gum elastic catheter was used as a permanent drainage-tube. After two or three days the cavity was washed out with the carbolic acid water, and several days after a weak solution of sulphate of zinc was used as a wash alternately with the carbolic acid.

At the end of six months the discharge was reduced to about a half ounce daily. In one year the patient had nearly recovered, the lung had expanded, but there was still a slight discharge of purulent matter from a small pocket which seemed to be gradually closing.

In the light of our experience it is fair to conclude, first, that, if in these cases the aspirator could have been used early, while effusion was yet serous, many months of suffering and great risks to life would have been prevented; and, second, that, in the late operation, only the free evacuation, permanent drainage, and antiseptic treatment were admissable.—*New York Medical Record.*

